# Consumption of various forms of apples is associated with a better nutrient intake and improved nutrient adequacy in diets of children: National Health and Nutrition Examination Survey 2003–2010

**DOI:** 10.3402/fnr.v59.25948

**Published:** 2015-10-05

**Authors:** Theresa A. Nicklas, Carol E. O'Neil, Victor L. Fulgoni

**Affiliations:** 1 Department of Pediatrics, Baylor College of Medicine, USDA/ARS Children's Nutrition Research Center, Houston, TX, USA; 2 School of Nutrition and Food Sciences, Louisiana State University Agricultural Center, Baton Rouge, LA, USA; 3 Nutrition Impact LLC, Battle Creek, MI, USA

**Keywords:** NHANES, apple, apple juice, applesauce, children, fruit, nutrient intake, nutrient adequacy

## Abstract

**Background:**

Consumption of fruit has been associated with a variety of health benefits, yet, 75% of children have usual intakes of total fruit below minimum recommended amounts. Apples are the second most commonly consumed fruit in the United States; however, no studies have examined the impact of apple consumption on nutrient intake and adequacy in children's diets.

**Objective:**

The purpose of this study is to examine the association between apple (various forms) consumption with nutrient intake and nutrient adequacy in a nationally representative sample of children.

**Design:**

Participants were children aged 2–18 years (*n=*13,339), from the National Health and Nutrition Examination Survey 2003–2010. Least square means of total energy and nutrient intake, and the percentage of the population below the estimated average requirement (EAR) or above the adequate intake (AI) among apple consumers and non-consumers were examined.

**Results:**

Consumers of total apple products had higher (*p*<0.01) total intakes of fiber, magnesium, and potassium and lower intakes of total fat, saturated fatty acids, monounsaturated fatty acid, and sodium than non-consumers. Apple consumers had higher (*p*<0.01) total sugar intake, but lower intake of added sugars compared to non-consumers. A lower (*p*<0.01) percentage of apple consumers were below the EAR for 13 of the 16 nutrients studied. Apple consumers had approximately a 10 percentage unit difference below the EAR for calcium and magnesium, and vitamins A, C, D, and E, than non-consumers. The percentage above the AI for fiber was significantly (*p*<0.0001) higher among total apple consumers (6.24±0.45 g) compared to non-consumers (0.57±0.07 g). The results were similar for individual apple products (i.e. apple juice, applesauce, and whole apples).

**Conclusion:**

Consumption of any forms of apples provided valuable nutrients in the diets of children.

Consumption of fruit has been associated with a variety of health benefits (1) including a reduced risk of cardiovascular disease (2–5), type 2 diabetes (5–7), and some types of cancer (5, 8). Despite these health benefits, approximately 75% of children (9–13 years of age) have usual intakes of total fruit below minimum recommended amounts (9). Most fruits are major contributors of a number of nutrients that are underconsumed in the United States, including dietary fiber, vitamins A and C, folate, and potassium. Dietary fiber and potassium have been identified as nutrients of public health concern by the 2010 Dietary Guidelines for Americans (DGA) (10). Most children consume dietary fiber and potassium in amounts lower than current recommendations (10).

One hundred percent fruit juice makes up approximately 35–50% of the total fruit intake of children 9–18 years (11) and contributes to their total nutrient intake and overall diet quality (12–15). Compared with non-consumers, consumers of 100% fruit juice had significantly higher intakes of energy, vitamins C and B_6_, riboflavin, iron, folate, magnesium, and potassium; and significantly lower intakes of saturated fatty acids (SFA), discretionary fat, and added sugars (12–14). Apples (*Malus domestica*) are the second most commonly consumed fruit in the United States (16), with 65% of the apple crop consumed as fresh fruit and 35% as processed apple products (e.g. juice, cider, or applesauce) (17). A medium raw apple (182 g), with skin, provides approximately 95 kilocalories (kcal), 19 g total sugars, 4 g dietary fiber (22% of the daily value), and 195 mg of potassium. In addition, raw apples contain virtually no total fat, SFA, sodium, or cholesterol. Processed apples have a slightly different nutrient profile than raw apples but still contribute valuable nutrients in the diet and count toward the fruit recommendation (10, 18). Apples are also the top source of phenolics, especially hydroxycinnamic acid derivatives and flavonoids (19) in the United States (20). There are no studies that have examined the impact of apple consumption (in various forms) on nutrient intake and nutrient adequacy in the diets of children. Thus, the purpose of this study is to examine the association between apple and apple product consumption (fresh fruit, apple sauce, or apple juice) with nutrient intake and nutrient adequacy in a nationally representative sample of children using the National Health and Nutrition Examination Survey (NHANES) 2003–2010 data.

## Subjects and methods

### Data collection

The NHANES is conducted on a continual basis by the National Center for Health Statistics of the Centers for Disease Control and Prevention. One of major objectives of NHANES is to provide data for investigators to be able to examine the relationship among diet, nutrition, and health (21). Details regarding the survey design, content, operations, and procedures are available online (21–23).

### Study population and dietary intake

Participants were children 2–18 years (*n=*13,339) who participated in the 2003–2010 NHANES. Demographic information was determined from the NHANES interview administered in the Mobile Examination Center. Intake data were obtained from What We Eat in America which collected an in-person, automated, multiple-pass, 24-hour dietary recall interview and a telephonic 24-hour dietary recall conducted 3–10 days later (24, 25). Detailed descriptions of the dietary interview methods are provided in the NHANES Dietary Interviewers Procedure Manuals (26, 27). Caretakers, usually parents, provided the 24-hour dietary recalls of children 2–5 years; children 6–11 years were assisted by an adult; all others provided their own recalls. Unreliable recall data as assessed by the USDA Food Surveys Research Group (*n=*275), pregnant and lactating females (*n=*83), and those consuming breast milk (*n=*10) were excluded from the analysis. The NHANES has stringent protocols and procedures that ensure confidentiality and protect individual participants from identification using federal laws (28) and additional Institutional Review Board approval was not required.

### Determination of apple product consumption

Apple and apple product consumption was determined by using the cycle-appropriate USDA Food and Nutrient Database for Dietary Studies food codes (29) for: 1) whole apples (food codes 62101100, 62101200, 62101220, 62101300, 63101000, 63101210, 63101310, 63101320, 63101330, 63101410, 63101420, 63101500, 63401060); 2) applesauce (which includes cooked apples) (food codes 63101110, 63101120, 63101130, 63101140, 63101150, 67102000, 67102010, 67102020, 67104060, 67104080); 3) 100% apple juice (food codes 64101010, 64104010, 64104050, 64104090, 67202000, 67202010); and 4) total apples (included all food codes from the first three groups). Although the term ‘whole apple’ is used in this study, this term refers not only to whole apples consumed out of hand but also includes apple slices. For each participant, daily total energy and nutrient intakes from foods and beverages were obtained from the total nutrient intake files associated with each data release. The Vitamin D Addendum to USDA Food and Nutrient Database for Dietary Studies 3.0 (29) was used to determine vitamin D intake. Intake from supplements was not considered. Several versions of the USDA Food and Nutrient Database for Dietary Studies were used to determine the nutrient content of foods in the four NHANES releases used in this study. Four separate apple consumption groups were generated: whole apples, applesauce, 100% apple juice, and total apples.

### Statistical analysis

Least square means (LSM) of total energy and nutrient intake were based on the first dietary recall data. The covariates used in the LSM analysis were age, gender, race/ethnicity, poverty income ratio grouped into three categories (<1.25, 1.25–3.49, and >3.49), physical activity level (sedentary, moderate, and vigorous), and total energy intake (the latter was not included in analysis of energy intake). The National Cancer Institute (NCI) Method (30) was used to estimate usual intake of selected nutrients in terms of nutrient adequacy. The 2 days of intake, using Day 1 sampling weights, were used to obtain necessary variance estimates. The NCI SAS (SAS Institute Inc., Cary, NC) macros Mixtran v.1.1 and Distrib v.1.1 were used to generate parameter estimates after covariate adjustment and to estimate the distribution of usual intake *via* the Monte Carlo method, respectively (31). Covariates for these analysis were sequence of participant's intake (Day 1 or Day 2) and a variable for weekday/weekend consumption. Differences among apple consumers and non-consumers among the four apple consumption groups were determined by computing population *Z* statistics generated from usual intake variables. The percentage of the population below estimated average requirement (EAR) or above the adequate intake (AI) (32) among apple consumers and non-consumers was also examined. For all analysis, study-specific dietary sample weights (33) were used to adjust the variance for the complex sample design of NHANES using the statistical package SUDAAN (version 9.0.3 [2007] Research Triangle Institute, Research Triangle Park, NC).

## Results

### Apple consumption and sample demographics

The sample consisted of children 2–18 years (*n=*13,339), of which ~26% of the population (*n=*3,482) consumed some form of apple products: ~12% (*n=*1,714) consumed apple juice, 3% (*n=*332) consumed applesauce, and 14% (*n=*1,891) consumed whole apple. There was overlap in the population, with some children consuming more than one apple product in 1 day. Among consumers, mean intake of any apple products was 222.2±3.9 g, apple juice was 9.6±0.24 fluid ounces (272.5±6.7 g; 1.2 cup equivalents), applesauce was 129.8±5.7 g (~1/2 cup equivalent), and whole apple was 143±3.8 g (~1 cup equivalent).

For the total sample, very few demographic differences were observed between consumers and non-consumers of total apple products. The non-consumers of apple products were significantly older (*p*<0.0001) with a higher percentage of current smokers (*p*<0.0001) compared to consumers (Table 1). Demographic differences for the specific apple product groups, namely apples, apple sauce, and apple juice are shown in Supplementary Table 1a–c. Compared to consumers, non-consumers of apple juice were significantly older (*p*<0.0001) with a higher percentage of current smokers (*p*<0.0001). Non-consumers of apple sauce were significantly older (*p*<0.0001) with a higher percentage of Mexican–Americans (*p*<0.0001) and a lower percentage of non-Hispanic whites (*p*<0.0001); a lower percentage reporting sedentary-to-moderate physical activity (*p*<0.01), and a higher percentage of current smokers (*p*<0.0001). Non-consumers of whole apple consumption were significantly older (*p*<0.0001) with a higher percentage of non-Hispanic blacks (*p*<0.01) and current smokers (*p*<0.0001), and a lower percentage of Mexican–Americans (*p*<0.01).

**Table 1:** Demographics of children 2–18 years (*n=*13,339) participating in NHANES 2003–2010 by total apple/apple product consumption

Variable	Consumers (*n=*3,482) LSM±SE	Non-consumers (*n=*9,857) LSM±SE	*P*
Gender (%)			
Female	49.85±1.43	48.95±0.82	0.5843
Ethnicity (%)			
Non-Hispanic Whites	58.25±1.97	60.97±2.01	0.3335
Non-Hispanic Blacks	13.01±1.10	14.88±1.09	0.2267
Mexican–American	15.93±1.27	12.38±1.17	0.0394
Age (years)	8.34±0.15	10.71±0.10	**<0.0001**
Poverty income ratio	2.56±0.07	2.49±0.06	0.4857
Physical activity (%)			
Sedentary	12.97±1.02	12.73±0.59	0.8385
Moderate	20.37±1.07	19.83±0.74	0.6756
Active	66.66±1.34	67.45±0.85	0.6206
Smoker, current (%)	2.45±0.38	7.46±0.48	**<0.0001**
Alcohol (g)	0.25±0.08	0.58±0.11	0.0122

LSM, least square means.

### Total apple consumption

Consumers of total apple products had significantly (*p*<0.01) higher total intakes of dietary fiber, magnesium, and potassium and lower intakes of total fat, SFA, monounsaturated fatty acids (MUFA), and sodium than non-consumers. Although consumers had a significantly higher total sugar intake, added sugar intake was lower compared to non-consumers (Table 2).

**Table 2:** Mean nutrient intake±SE in children 2–18 years participating in NHANES 2003–2010 by total apple and apple juice consumption (*n=*13,339)

	Total apple			Apple juice		
		
Variables^a^,^b^	Consumers (*n=*3,482) LSM±SE	Non-consumers (*n=*9,857) LSM±SE	*P*	Consumers (*n=*1,714) LSM±SE	Non-consumers (*n=*11,625) LSM±SE	*P*
Energy (kcal)	2033.84±23.00	1975.85±13.51	0.0297	2048.84±26.66	1982.65±12.33	0.0242
Protein (g)	68.93±0.62	69.98±0.35	0.1408	68.17±0.73	69.93±0.33	0.0277
Total sugar (g)	142.51±1.37	132.62±0.97	**<0.0001**	144.19±1.61	133.90±0.92	**<0.0001**
Added sugar (tsp eq)	18.22±0.29	21.61±0.25	**<0.0001**	17.44±0.38	21.20±0.24	**<0.0001**
Dietary fiber (g)	14.97±0.19	12.22±0.11	**<0.0001**	13.25±0.22	12.89±0.12	0.1563
Total fat (g)	70.63±0.51	74.51±0.33	**<0.0001**	70.82±0.64	73.89±0.32	**<0.0001**
SFA (g)	24.64±0.21	26.32±0.15	**<0.0001**	24.67±0.32	26.06±0.13	**<0.0001**
MUFA (g)	25.48±0.25	27.24±0.16	**<0.0001**	25.57±0.29	26.95±0.15	**<0.0001**
PUFA (g)	14.48±0.20	14.62±0.13	0.5768	14.48±0.26	14.60±0.12	0.6740
Cholesterol (mg)	216.03±5.67	221.59±2.47	0.3682	225.06±9.35	219.46±2.22	0.5600
Vitamin A, RAE (µg)	612.17±14.93	570.08±7.38	0.0115	583.38±17.57	580.62±7.45	0.8851
Vitamin D (µg)	6.06±0.17	5.72±0.08	0.0649	5.70±0.20	5.82±0.08	0.5604
Folate, DFE (µg)	532.04±9.92	526.58±6.17	0.6400	511.59±10.48	530.29±6.19	0.1244
Total choline (mg)	251.70±4.41	248.58±2.05	0.5216	250.28±6.61	249.28±2.14	0.8850
Calcium (mg)	1043.89±14.80	1008.51±8.79	0.0399	1017.74±20.16	1017.66±8.06	0.9970
Magnesium (mg)	242.53±2.36	225.81±1.31	**<0.0001**	231.19±2.44	229.98±1.39	0.6677
Iron (mg)	14.73±0.18	14.27±0.10	0.0255	14.68±0.28	14.35±0.10	0.2571
Sodium (mg)	3079.31±23.31	3172.41±20.01	**0.0024**	3073.21±32.36	3158.98±18.21	0.0209
Potassium (mg)	2407.70±28.50	2148.72±15.72	**<0.0001**	2398.94±33.65	2189.71±16.65	**<0.0001**
Phosphorus (mg)	1270.05±11.97	1251.69±7.20	0.1887	1250.80±15.82	1257.23±6.60	0.7072

Data source: Participants 2–18 years of age of the NHANES 2003–2010.

LSM, least square means.

a Covariates for energy: gender, ethnicity, age, poverty income ratio, and physical activity.

b Covariates for nutrients: gender, ethnicity, age, poverty income ratio, physical activity, and energy (kcals).

### Apple juice consumption

Consumers of apple juice had significantly (*p*<0.01) higher intakes of potassium and lower intakes of total fat, SFA, and MUFA. Although the apple juice consumers had a significantly higher intake of total sugars, added sugar intake was lower compared to non-consumers. Dietary fiber and sodium were similar for consumers and non-consumers (Table 2).

### Applesauce consumption

Consumers of applesauce had significantly (*p*<0.01) higher intakes of total sugars than non-consumers. No other nutrient intake differences were found among the consumers and non-consumers of applesauce (Table 3).

**Table 3:** Mean nutrient intake±SE in children aged 2–18 years participating in NHANES 2003–2010 by applesauce and whole apple consumption (*n*=13,339)

	Applesauce			Whole apple		
		
Variables^a^,^b^	Consumers (*n=*332) LSM±SE	Non-consumers (*n=*13,007) LSM±SE	*P*	Consumers (*n=*1,891) LSM±SE	Non-consumers (*n=*11,448) LSM±SE	*P*
Energy (kcal)	1997.93±46.30	1990.53±12.19	0.8773	2052.65±32.88	1980.65±12.41	0.0405
Protein (g)	70.02±1.22	69.70±0.32	0.8045	69.73±0.78	69.71±0.35	0.9794
Total sugar (g)	144.99±3.79	134.86±0.85	**0.0092**	140.93±2.09	134.23±0.91	**0.0033**
Added sugar (tsp eq)	19.45±0.78	20.78±0.23	0.1043	18.02±0.40	21.18±0.23	**<0.0001**
Dietary fiber (g)	13.71±0.30	12.91±0.12	0.0146	16.96±0.23	12.28±0.11	**<0.0001**
Total fat (g)	70.51±1.30	73.60±0.28	0.0205	70.28±0.73	74.03±0.30	**<0.0001**
SFA (g)	25.15±0.59	25.91±0.13	0.2094	24.47±0.29	26.12±0.14	**<0.0001**
MUFA (g)	25.50±0.59	26.82±0.13	0.0282	25.31±0.37	27.02±0.14	**<0.0001**
PUFA (g)	13.78±0.33	14.61±0.11	0.0167	14.53±0.27	14.59±0.12	0.8462
Cholesterol (mg)	226.28±13.41	219.97±2.34	0.6429	210.94±6.67	221.66±2.58	0.1338
Vitamin A, RAE (µg)	621.10±33.31	579.70±7.84	0.2264	641.40±18.99	571.12±7.52	**0.0006**
Vitamin D (µg)	6.17±0.33	5.80±0.08	0.2683	6.33±0.21	5.72±0.08	**0.0077**
Folate, DFE (µg)	503.82±22.59	528.75±5.65	0.2844	554.52±13.17	523.65±5.85	0.0322
Total choline (mg)	259.74±10.97	249.04±2.16	0.3385	253.92±4.88	248.64±2.16	0.3223
Calcium (mg)	1068.61±29.58	1016.05±8.15	0.0867	1063.70±19.83	1010.16±8.59	0.0132
Magnesium (mg)	231.57±3.78	230.09±1.38	0.7120	254.38±3.42	226.18±1.34	**<0.0001**
Iron (mg)	14.28±0.43	14.40±0.09	0.8005	14.93±0.22	14.30±0.10	**0.0096**
Sodium (mg)	3134.84±69.94	3148.79±17.12	0.8464	3073.04±36.42	3160.67±18.64	0.0322
Potassium (mg)	2299.86±52.19	2212.96±17.33	0.1141	2465.03±37.36	2174.93±16.32	**<0.0001**
Phosphorus (mg)	1274.23±22.56	1255.87±6.89	0.4363	1289.66±15.41	1251.02±7.19	0.0231

Data source: Participants 2–18 years of age of the NHANES 2003–2010.

a Covariates for energy: gender, ethnicity, age, poverty income ratio, and physical activity.

b Covariates for nutrients: gender, ethnicity, age, poverty income ratio, physical activity, and energy (kcals).

### Whole apple consumption

Consumers of whole apples had significantly (*p*<0.01) higher intakes of dietary fiber, magnesium, potassium, iron, and lower intakes of total fat, SFA, and MUFA than non-consumers. Although whole apple consumers had significantly higher intake of total sugars, added sugar intake was lower than non-consumers (Table 3).

### Percentage of children with usual intakes not meeting recommendations

**Fig. 1:**
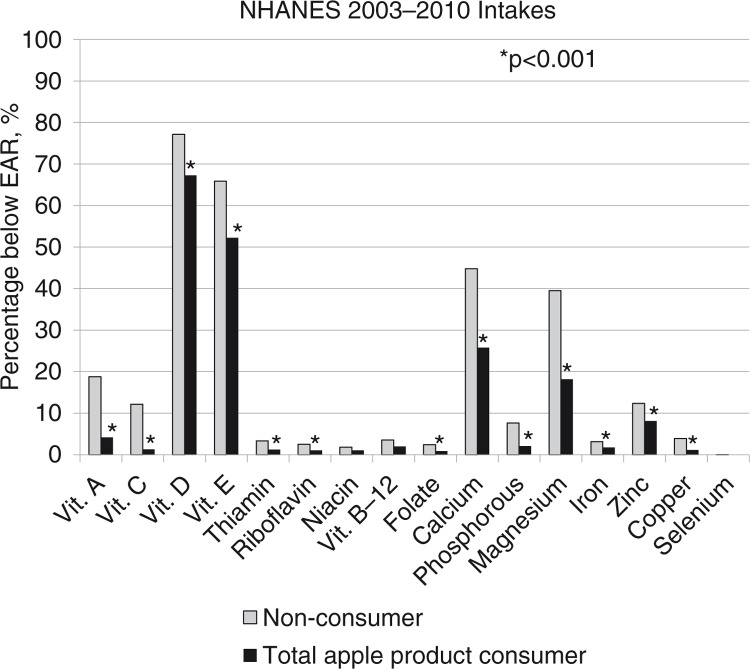
Percentage of children with usual intakes not meeting recommendations by total apple consumption.

A significantly (*p*<0.01) lower percentage of total apple consumers was below the EAR for 13 of the 16 nutrients studied (Fig. 1). Total apple consumers had approximately a 10 percentage unit difference in the population below the EAR for calcium and magnesium, and vitamins A, C, D, and E. The percentage above the AI for fiber was significantly (*p*<0.0001) higher among total apple consumers (6.24±0.45) compared to non-consumers (0.57±0.07). No differences were found in the percentage above the AI for potassium (data not shown). The results were very similar for the specific apple products (i.e. apple juice, applesauce, and whole apple) (data not shown).

## Discussion

This is the first published study to examine apple and apple product intake and their effects on the nutrient intake and dietary adequacy of children. Approximately, 26% of the population consumed some type of apple product. Whole apple, apple juice, or total apple consumers consumed an average of at least one cup equivalent of the corresponding product on the day of the recall. Applesauce consumers consumed approximately half that amount. Only applesauce was lower than the number of cup equivalents seen in a national study that examined all whole fruit and 100% fruit juice consumption of children 2–18 years (34), suggesting that apple consumption made a major contribution to the total fruit intake by children.

The fruit recommendation for children is age and gender dependent with 1.5 or 2 cups as the recommendation for most children over 4 years (18). Intake of fruit, including 100% fruit juice, is important because it has been associated with increased nutrient intake and adequacy (35–37), which in turn, is associated with reduced risk of chronic disease (2–5, 38, 39). The majority of children do not meet the recommendation for fruit (40, 41). Apple consumers have a higher intake of total fruit than non-consumers (data not shown) suggesting not only apples contribute to increased fruit consumption, but apple consumption may also be associated with overall increased fruit intake, as has been shown in studies looking at consumption of 100% fruit juice (12, 13, 42).

Consumers of total apple products had significantly higher total intakes of dietary fiber, magnesium, and potassium than non-consumers. Dietary fiber and potassium were identified as nutrients of public health concern and magnesium is a shortfall nutrient identified by the 2010 DGA (10). Consumers also had higher mean intakes of total sugars than non-consumers, however, levels of added sugars were lower in every consumption group except those consuming applesauce, suggesting that the nutrient intakes are reflecting the intrinsic sugars of the apples. Applesauce can be prepared with or without added sugars (43), so this may account for the differences shown in mean intakes of total sugars and added sugars. Finally, consumers had lower mean intakes of two nutrients to limit – SFA and sodium than non-consumers. Total apple consumption was also associated with improved nutrient adequacy for 15 of the 20 nutrients studied, specifically for vitamins A, C, D, and E; calcium; magnesium; and dietary fiber. A full discussion of all these nutrients is beyond the scope of this paper, but it is important to highlight shortfall nutrients and nutrients to limit (10).

Unlike apple juice and applesauce consumers, whole apple and apple product consumers had higher mean intakes and better dietary adequacy for dietary fiber when compared with non-consumers. This was not surprising because whole apples are a good source of dietary fiber (44) and apple juice and applesauce are low in dietary fiber (43). Dietary fiber recommendations for children vary, so comparison with standards can be difficult (45). Mean intakes of dietary fiber in consumers in this study, using the Dietary Reference Intakes (46): 14 g/d and 25 g/d for children 1–3 and 4–8 years, respectively; 31 g/d and 38 g/d for males 9–13 and 14–18 years, respectively; and 26 g/d for females 9–18 years, were still lower than the recommended amounts.

Apples (with skin) contain a mix of insoluble (3.1 g/1 medium apple) and soluble (1.3 g/1 medium apple) fiber (47, 48). Apples are an especially rich source of pectin (49), which is a complex set of plant, non-starch polysaccharides (50). Apples should be consumed with their peels because discarding the peels reduces the fiber content by up to 25% (51). In children, fiber intake is inversely associated with blood cholesterol levels (52–55) and constipation (45, 56–58), which is a major cause of morbidity (59). The purported mechanisms behind the health effects of fiber are not well established; benefits from fiber may result from changes in intestinal viscosity, rate of passage of intestinal contents, nutrient absorption, and short-chain fatty acids and gut hormone production (60). Thus, fiber-dense foods, including whole apples, should be encouraged among children.

Although all groups of apple consumers, except applesauce consumers, had higher mean intakes of potassium, a nutrient of public health concern, these groups did not have better nutrient adequacy values when compared with non-consumers. The reasons are likely twofold: apples do have some potassium (43), however, they are not considered a potassium-rich fruit, like bananas or oranges, and the potassium recommendation is relatively high (10, 61) and intake is relatively low (62, 63); thus, it is difficult to make a significant difference in nutrient adequacy.

Only whole apple and total apple consumers had higher intakes of magnesium and better dietary adequacy than seen in non-consumers; neither applesauce nor apple juice showed this pattern. None of the apple products is a rich source of magnesium, so it is likely that the higher intakes of this mineral are not the result of apple product consumption, *per se*, but rather foods that may be consumed with apples, for example, nuts (43). This was not part of this study but does represent a further line of research, for example, examining apple dietary patterns and how apple consumption influences consumption of other foods.

Of the nutrients to limit, added sugars were lower in all groups except applesauce consumers, as discussed above. In all consumption groups except the applesauce consumers, SFA intake was lower than that in non-consumers. Because applesauce has virtually no fat added (43), this finding likely reflects foods consumed with applesauce, such as roasted meats, or other foods consumed during the rest of the day. On average, however, all four groups of consumers and non-consumers exceeded the recommendation for SFA of less than 10% of energy (10). Sodium intake was lower in the total apple consumption group only; although it was higher than recommendations in all groups (10). It is important when interpreting nutrient differences in a 24-hour period that these differences do not reflect apple consumption solely but reflect other foods consumed throughout the day. It has been shown that children who consume apple products do have overall better diet quality (64).

Of special interest were the results from the 100% apple juice consumption group. Apple juice consumption, in particular, has been associated with not only obesity in children, but also with stunted growth in children 2 and 5 years (65); although another study was unable to replicate their findings (64). In contrast with other studies looking at consumption of 100% fruit juice (12, 13), this study did not show higher energy intake among apple juice consumers when compared with non-consumers. Furthermore, the high sugar content of 100% fruit juice has been cited as a reason to limit consumption. This study showed that although total sugars were higher in apple juice consumers they were also higher in all groups of apple consumers and added sugars were not higher. Unfortunately 100% fruit juice consumption is declining (34) and children are missing the opportunity for greater intake of important nutrients, such as potassium. Aside from potassium, intake of none of the other micronutrients was lower in 100% apple juice consumers, suggesting that 100% apple juice, even when consumed in greater amounts than recommended by the American Academy of Pediatrics for young children (66), does not lead to inadequate intakes of micronutrients or excess energy intake.

## Strengths and limitations

The strengths of this study were that it included a large sample size with a nationally representative sample of children. The NHANES has carefully controlled protocols and screens 24-hour dietary recalls confirming they are valid and complete; the NHANES also uses the multiple-pass method to obtain dietary intake, which is the best dietary assessment method available for large-scale, epidemiologic studies. The assessment of nutrient adequacy in this study is an improvement over other studies of fruit that have simply examined nutrient intake (12, 67), because it is a better estimate of how groups meet the EAR and AI than mean nutrient intake. To determine nutrient adequacy, an individual's usual intake (UI) must be determined, so more than one dietary recall must be available. Twenty-four-hour dietary recalls used in this study do have several intrinsic limitations: they are memory dependent, and under- and over-reporting may occur. In proxy-assisted recalls of children, parents may know what their children consume at home (68, 69), but they may not know what their children consume outside the home, for example in school or day care (70). The sample size for children consuming applesauce was small, so one needs to be cautious when interpreting the results. Finally, cause-and-effect relationships cannot be determined from cross-sectional, epidemiologic data.

## Conclusion and recommendations

In conclusion, the consumption of whole apples, applesauce, apple juice, and all apple products provided valuable nutrients in the diets of children. All apple products should be encouraged, in age-appropriate amounts and as part of a healthy diet (71) in children to help them meet nutrient requirements.

## Supplementary Material

Consumption of various forms of apples is associated with a better nutrient intake and improved nutrient adequacy in diets of children: National Health and Nutrition Examination Survey 2003–2010

Consumption of various forms of apples is associated with a better nutrient intake and improved nutrient adequacy in diets of children: National Health and Nutrition Examination Survey 2003–2010

Consumption of various forms of apples is associated with a better nutrient intake and improved nutrient adequacy in diets of children: National Health and Nutrition Examination Survey 2003–2010
