# Supplementary material for: Consumption of various forms of apples is associated with a better nutrient intake and improved nutrient adequacy in diets of children: National Health and Nutrition Examination Survey 2003–2010
Source: Food Nutr Res. 2015 Oct 5;59:10.3402/fnr.v59.25948. doi: 10.3402/fnr.v59.25948 (PMC4595465; doi:10.3402/fnr.v59.25948)
Supplement: Consumption of various forms of apples is associated with a better nutrient intake and improved nutrient adequacy in diets of children: National Health and Nutrition Examination Survey 2003–2010 [file FNR-59-25948-s001.pptx]

## Slide 1
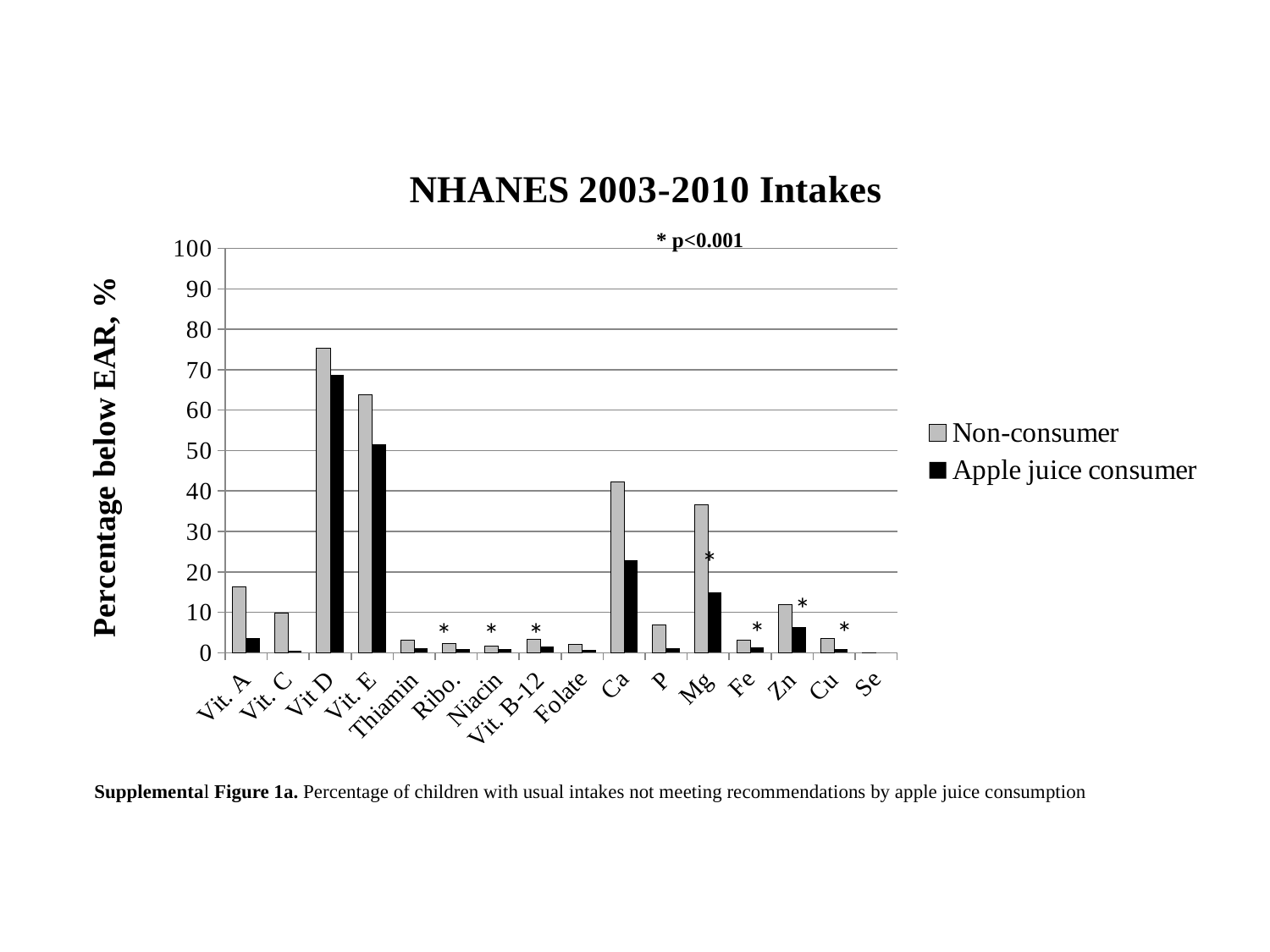

### Chart: NHANES 2003-2010 Intakes
| Category | Non-consumer | Apple juice consumer |
|---|---|---|
| Vit. A | 16.2 | 3.58 |
| Vit. C | 9.82 | 0.45 |
| Vit D | 75.38 | 68.65 |
| Vit. E | 63.8 | 51.53 |
| Thiamin | 3.03 | 1.07 |
| Ribo. | 2.26 | 0.92 |
| Niacin | 1.72 | 0.96 |
| Vit. B-12 | 3.37 | 1.5 |
| Folate | 2.14 | 0.62 |
| Ca | 42.28 | 22.84 |
| P | 6.94 | 1.14 |
| Mg | 36.64 | 15.02 |
| Fe | 3.02 | 1.33 |
| Zn | 11.96 | 6.35 |
| Cu | 3.5 | 0.93 |
| Se | 0.02 | 0.0 |Supplemental Figure 1a. Percentage of children with usual intakes not meeting recommendations by apple juice consumption
