# Supplementary material for: Consumption of various forms of apples is associated with a better nutrient intake and improved nutrient adequacy in diets of children: National Health and Nutrition Examination Survey 2003–2010
Source: Food Nutr Res. 2015 Oct 5;59:10.3402/fnr.v59.25948. doi: 10.3402/fnr.v59.25948 (PMC4595465; doi:10.3402/fnr.v59.25948)
Supplement: Consumption of various forms of apples is associated with a better nutrient intake and improved nutrient adequacy in diets of children: National Health and Nutrition Examination Survey 2003–2010 [file FNR-59-25948-s002.pptx]

## Slide 1
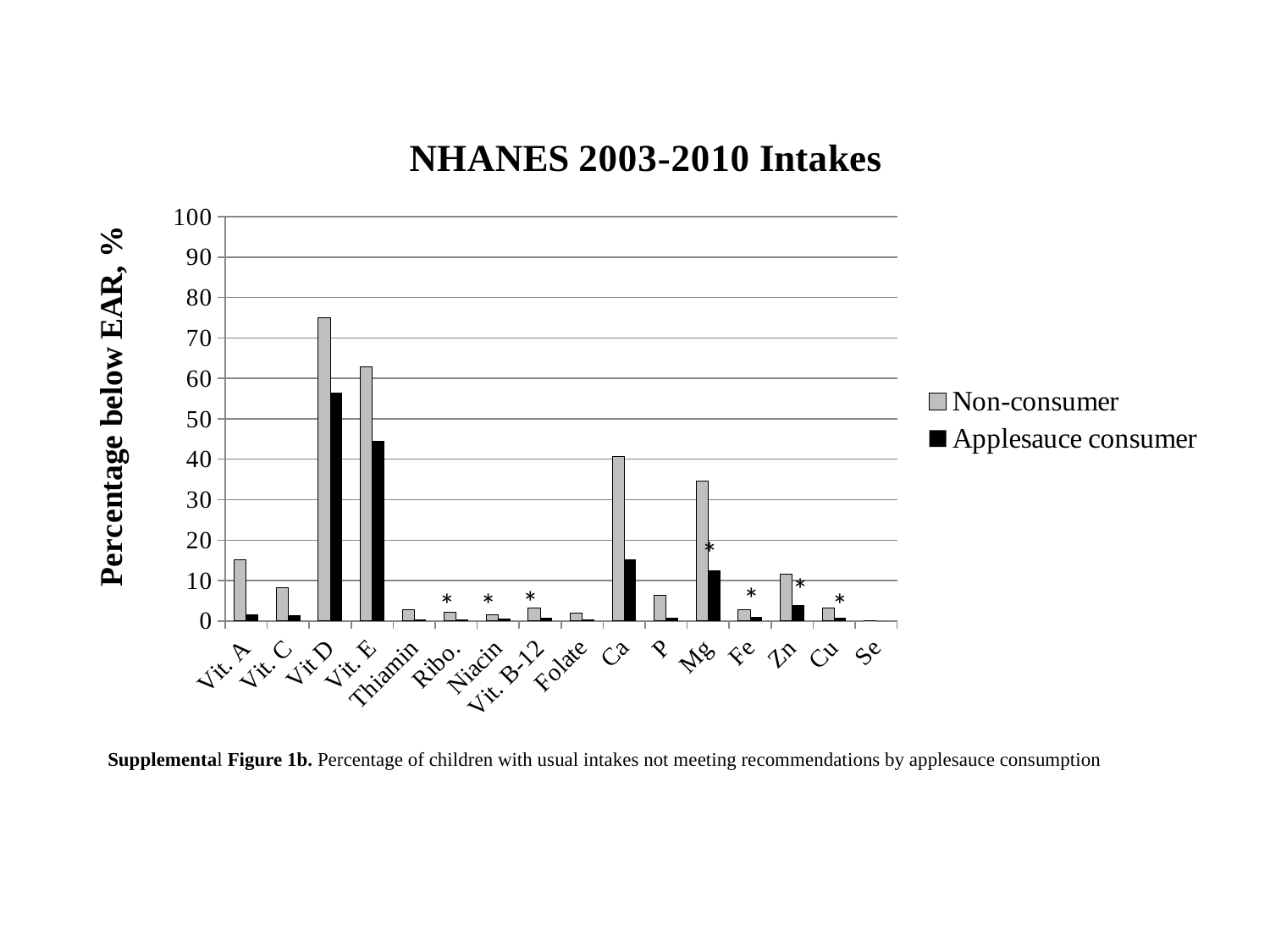

### Chart: NHANES 2003-2010 Intakes
| Category | Non-consumer | Applesauce consumer |
|---|---|---|
| Vit. A | 15.21 | 1.62 |
| Vit. C | 8.32 | 1.47 |
| Vit D | 74.98 | 56.54 |
| Vit. E | 62.89 | 44.6 |
| Thiamin | 2.89 | 0.44 |
| Ribo. | 2.19 | 0.48 |
| Niacin | 1.63 | 0.63 |
| Vit. B-12 | 3.25 | 0.82 |
| Folate | 2.02 | 0.33 |
| Ca | 40.69 | 15.21 |
| P | 6.26 | 0.87 |
| Mg | 34.57 | 12.57 |
| Fe | 2.83 | 0.98 |
| Zn | 11.62 | 4.0 |
| Cu | 3.22 | 0.78 |
| Se | 0.02 | 0.0 |Supplemental Figure 1b. Percentage of children with usual intakes not meeting recommendations by applesauce consumption
