# Supplementary material for: Consumption of various forms of apples is associated with a better nutrient intake and improved nutrient adequacy in diets of children: National Health and Nutrition Examination Survey 2003–2010
Source: Food Nutr Res. 2015 Oct 5;59:10.3402/fnr.v59.25948. doi: 10.3402/fnr.v59.25948 (PMC4595465; doi:10.3402/fnr.v59.25948)
Supplement: Consumption of various forms of apples is associated with a better nutrient intake and improved nutrient adequacy in diets of children: National Health and Nutrition Examination Survey 2003–2010 [file FNR-59-25948-s003.pptx]

## Slide 1
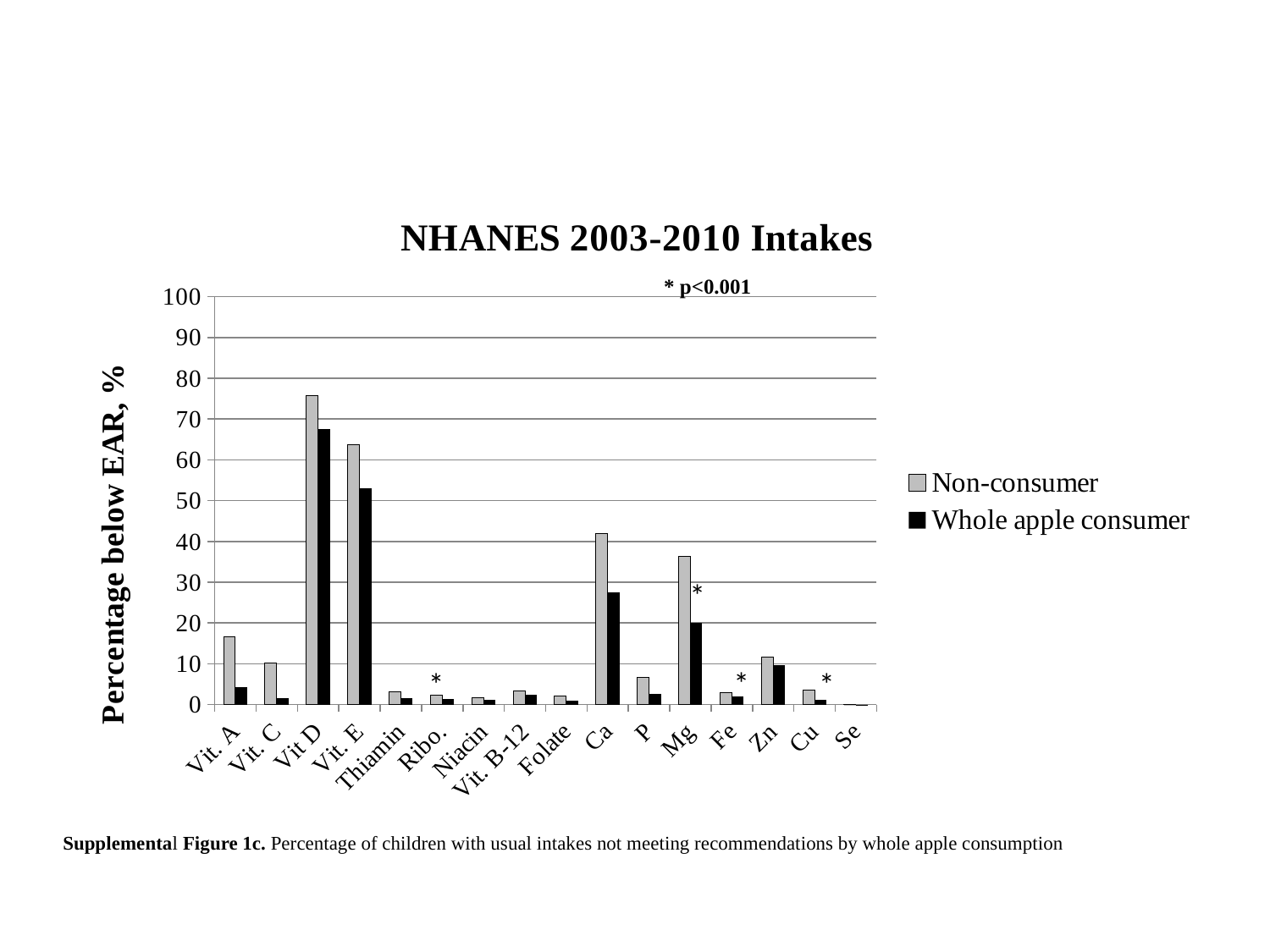

### Chart: NHANES 2003-2010 Intakes
| Category | Non-consumer | Whole apple consumer |
|---|---|---|
| Vit. A | 16.67 | 4.36 |
| Vit. C | 10.11 | 1.49 |
| Vit D | 75.77 | 67.48 |
| Vit. E | 63.81 | 53.06 |
| Thiamin | 3.03 | 1.58 |
| Ribo. | 2.25 | 1.27 |
| Niacin | 1.68 | 1.15 |
| Vit. B-12 | 3.28 | 2.39 |
| Folate | 2.18 | 0.97 |
| Ca | 41.97 | 27.42 |
| P | 6.75 | 2.64 |
| Mg | 36.37 | 19.98 |
| Fe | 2.92 | 1.99 |
| Zn | 11.57 | 9.62 |
| Cu | 3.54 | 1.25 |
| Se | 0.02 | 0.01 |Supplemental Figure 1c. Percentage of children with usual intakes not meeting recommendations by whole apple consumption
